# Nanoporous foam fabricated by dealloying AgAl thin film through supercritical fluid corrosion

**DOI:** 10.1039/c8ra00463c

**Published:** 2018-04-09

**Authors:** Y. C. Liu, J. C. Huang, X. Wang, M. T. Tsai, Z. K. Wang

**Affiliations:** Department of Materials and Optoelectronic Science, National Sun Yat-Sen University Kaohsiung Taiwan Republic of China jacobc@mail.nsysu.edu.tw chihuang@cityu.edu.hk +886-7-525-4099 +886-7-525-2000 ext. 4063; Institute for Advanced Study, Department of Materials Science & Engineering, City University of Hong Kong Kowloon Hong Kong; School of Mechanical Engineering, Liaoning Shihua University Fushun P. R. China

## Abstract

In this research, nanoporous silver foams are fabricated through dealloying Ag_35_Al_65_ (as atomic percentage, at%) thin films in supercritical (SC) carbon dioxide. The supercritical CO_2_ is mixed with either HCl, water or H_2_C_2_O_4_ aqueous solution as the solute in the reaction chamber. Due to the low tension of the supercritical fluid, under the best operating conditions, the surface area of the as-dealloyed Ag_35_Al_65_ can reach 4.6 m^2^ g^−1^, and the porosity volume fraction value can reach 74%, with a smallest average pore size of around 75 nm. In an optimum supercritical CO_2_ environment, a lower chemical concentration can be applied and it can take less time to form a uniform nanoporous structure.

## Abstract 2

In this research, nanoporous silver foams are fabricated through dealloying Ag_35_Al_65_ (as atomic percentage, at%) thin films in supercritical (SC) carbon dioxide.

## Graphical abstract

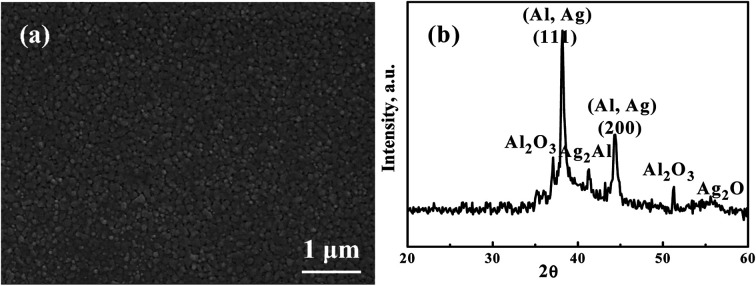

## 1. Introduction

Porous materials have existed in nature for a long time and could be used in many areas because of their low density, small pores, and high surface area. Because of their high specific area and certain special properties, nanoporous materials have attracted more and more attention in many fields, such as catalysis,^1–5^ optics,^6^ sensing,^7^ and so on.

Chemical dealloying^8^ has been widely used to prepare nanoporous noble metals; it is a corrosion process using an acid or alkali solution to react with alloys. This process is an ancient technology and is also known as the selective dissolution of the more active element. The advantages of chemical dealloying are that it is cheap, simple and useful, which are the main reasons why it has been applied to fabricating nanoporous materials for a few decades. This process results in the formation of a nanoporous sponge composed almost entirely of the nobler alloy constituent. During the period that the less noble component is dissolved away from the precursor alloy, the remaining nobler element simultaneously diffuses and agglomerates into a well-defined 3D bi-continuous nanoporous structure.

Over the past years, dealloying has been mostly performed using the prototypical Au–Ag system to fabricate nanoporous Au (NPG). In 2001, Erlebacher *et al.*^8^ used chemical dealloying to fabricate NPG from an Au–Ag system, which is recognized as the first nanoporous material made using the chemical dealloying method. The ligament spacing is about 5 nm to 10 nm and an open channel microstructure exists. Recently, many other alloy systems have been studied in order to form nanoporous materials, such as Au–Ag,^8^ Ag–Cu,^9^ Ag–Al,^2,10–13^ Cu–Zr,^14,15^ Al–Cu,^16^*etc.* Of all the alloy systems, the Ag–Al alloy has received attention as the more efficient dealloying system for developing nanoporous Ag materials for various applications.^17–19^ Using the Ag–Al system, chemical dealloying of Ag–Al alloys can be done in acidic or alkaline aqueous solution under free corrosion conditions.^2,10–13^ Using 5 wt% HCl as the dealloying solution, a composition of from 15 to 40 at% of Ag in the precursor alloy can be dealloyed.^2,11,12^ If 5 wt% HCl is replaced by 20 wt% NaOH, Ag_2_Al will still remain after dealloying.^10^ This result shows that using a 5 wt% HCl dealloying solution has a better effect than using alkaline solution for selective corrosion with Ag and Al. The typical chemical dealloying processes mentioned above usually require an elevated temperature (60–120 °C) and longer time (60–150 min). For example, Al–Ag ribbons can be dealloyed in 5 wt% HCl aqueous solution first at 60 °C for 30 minutes until no obvious bubbles emerge, and it then takes 120 min for the same solution at 90 °C to reach a dealloying state.^11^

Supercritical fluids (SCFs) offer the possibility to obtain new products using their unique characteristics, which are environmentally friendly and sustainable.^20–22^ SCFs are very dense gases with many properties superior to liquids or solvents. When a substance turns from a liquid or gas into a SCF, such a SCF would have a diffusion rate higher than that of the liquid and a viscosity ratio similar to that of the gas. For instance, the diffusion rate of liquid H_2_O is smaller than 10^−5^ cm^2^ s^−1^, with a viscosity ratio near 10^−2^ g cm^−1^ s^−1^, while supercritical water (SCW) has a diffusion rate of about 10^−2^ to 10^−5^ cm^2^ s^−1^ with a viscosity ratio of 10^−3^ to 10^−6^ g cm^−1^ s^−1^.^23^ The high diffusion rate provides a high diffusion flux in a SCF. Because almost all SCFs need to be formed at higher temperatures, the diffusion coefficient could increase a lot. Besides, a low viscosity ratio would let SCFs have strong and high penetration. With the above advantages, SCFs are recognized as an “ideal solution”.^21^ Health and safely benefits are especially evident in the use of some of the most important SCFs, supercritical CO_2_ (SC CO_2_ or SCC) and supercritical H_2_O (SCW). They are non-carcinogenic, nontoxic, non-mutagenic, non-flammable and thermodynamically stable.^21^ In this article, we will use SC CO_2_ as the solution for chemical dealloying.

In 2012, Ruhl *et al.*^24^ demonstrated corrosion in supercritical CO_2_*via* the diffusion of flue gas acids or water. Hydrochloric acid in SC CO_2_ is very reactive and aggressive, including towards the austenitic autoclave material. Later in 2013, Morrish *et al.*^25^ used hexafluoroacetylacetone (hfacH) and H_2_O_2_ dissolved in supercritical CO_2_ to fabricate nanoporous copper. The good oxidation ability of H_2_O_2_ made CuO appear, and hfacH dissolved CuO in order to form a nanoporous structure. Ag–Cu alloys exhibit eutectic phase behavior, allowing the composition of the two phases to be fixed while varying the average size of the phase domains from 250 to 1000 nm, through increasing the annealing temperature. The selective removal of Cu from both phases was observed, and higher concentrations of the etching solution increased the etching rate between 45 and 75 °C.^25^

Water has high solubility in SC CO_2_,^26^ and both water and CO_2_ are common materials we can obtain from nature. Normal reverse osmotic water has carbonate (CO_3_^2−^) inside, because a small amount of CO_2_ gas in the atmosphere dissolves in water. The water has strong oxidative capacity in SC CO_2_, and could react with Al. The alumina ion will hydrolyze because of carbonate, and form aluminum carbonate. Aluminum carbonate will form aluminum hydroxide after reacting with water. With SC CO_2_ mixed with water, we could dealloy an Ag–Al alloy.

From the research mentioned above, using SC CO_2_ as the solvent to fabricate a nanoporous material can be regarded as an improvement over conventional chemical dealloying methods. It can not only use less acid to produce porous material, but can also accomplish the process in a shorter time. The high penetration and high solubility of supercritical fluids give the acid solute in SC CO_2_ stronger oxidant ability, causing the chemical reaction rate to be much higher. Thus, this research examines the performance of dealloying in supercritical CO_2_ in comparison to conventional chemical dealloying.

## 2. Experimental procedures

In this study, AgAl thin films were prepared *via* sputtering deposition, using DC guns. As the two targets are both metals (silver and aluminum), it is more efficient to use DC sputtering. To ensure the quality of the thin films, the base pressure of the operating chamber was first set to 5 × 10^−7^ torr. Then Ar was chosen to be the working gas. Both the Ag and Al guns had a pure elemental target with 99.99% purity. The Al and Ag guns were adjusted to 85 W and 15 W, respectively. During deposition, the substrate was rotated with an average speed of 15 rpm and the working distance was 120 mm. All prepared thin films have a film thickness of 400–500 nm. To protect the aluminum from severe oxidation (Al_2_O_3_) after the sputtering process, we smeared a photoresist upon the thin film. The photoresist was composed of dimethyl sulfoxide (DMSO), so it could be easily dissolved using acetone. The resulting thin film composition is Ag_35_Al_65_ (at%) within ±1 at% accuracy.

Firstly, we used Ag_35_Al_65_ to observe the differences between dealloying with HCl, water, and H_2_C_2_O_4_ in supercritical CO_2_. The supercritical CO_2_ pressure level was tried at 1200, 1600 and 2000 psi, and it is consistently found that the highest pressure of 2000 psi can result in a higher degree of dealloying. In parallel, the supercritical CO_2_ temperature was tried at 60, 90 and 120 °C, and 120 °C consistently yields better efficiency. Thus, in this study, the SCF pressure and temperature are fixed at 2000 psi and 120 °C.

Then, the Ag_35_Al_65_ thin films were dealloyed through supercritical fluid mixed with 0.05 ml of 5 wt% or 1 M HCl, 5 ml of water, or 0.25 ml of 5 wt% H_2_C_2_O_4_. The samples were characterized *via* different kinds of measurements. To compare the differences between dealloying in supercritical CO_2_ and conventional chemical dealloying, conventional chemical dealloying routines using HCl, H_2_C_2_O_4_ and even water were also conducted in this study. Note that conventional chemical dealloying with water alone would not result in any corrosion.

The as-dealloyed Ag–Al thin films were examined *via* X-ray diffraction (XRD), and the morphology and composition were identified using scanning electron microscopy (SEM) with energy dispersive X-ray spectrometry (EDS). The surface area and volume porosity of the as-dealloyed samples were evaluated *via* the Brunauer, Emmet and Teller (BET) test and ImageJ software. For the BET experiments, the first step of this process is to measure the weight of the sample and the weight of the sample with the glass tube. Then a process of degasification was carried out in order to get rid of the vapor existing inside the sample. The weights of the sample and glass tube were measured again in order to calculate the weight of the vapor. The weight of the sample minus the weight of the vapor equals the true weight of the sample. Then, the sample and the glass tube were put under an environment full of liquid nitrogen, and then the nitrogen gas filled the glass tube. The measurements of the ratio of surface area and the ratio of pore volume are carried out for 8 h.

## 3. Results and discussion

### Thin film before dealloying

Based on the Al–Ag equilibrium phase diagram,^27^ there exists a β-Ag_2_Al phase (of hexagonal close packed HCP structure) for an Al content of around 25 to 40 at%, and there is a eutectic point at around Ag_34_Al_66_ (at%) for face-centered cubic (FCC) α-Al and HCP β-Ag_2_Al. The composition of the present Ag_35_Al_65_ thin films would reside within the two-phase region, namely, FCC pure α-Al + HCP β-Ag_2_Al.

**Fig. 1:**
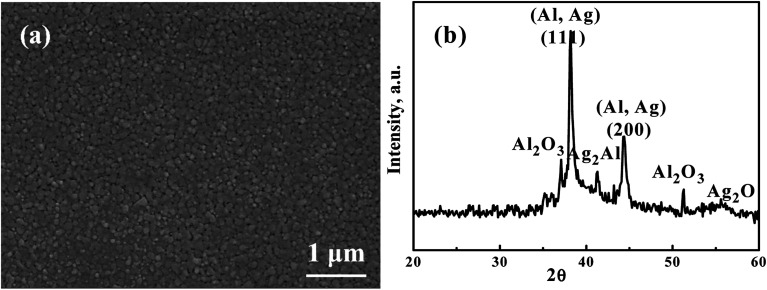
A representative (a) SEM secondary electron image (SEI) micrograph and (b) XRD pattern of the as-sputtered Ag_35_Al_65_ films before dealloying.

Fig. 1(a) presents a representative SEM secondary electron image (SEI) micrograph of the as-sputtered Ag_35_Al_65_ film before dealloying. The composition is confirmed to be Ag_35_Al_65_ (at%) within ±1 at% accuracy. The XRD pattern of the as-deposited Ag_35_Al_65_ film before dealloying is shown in Fig. 1(b). There is always pure Al (and/or some minor pure Ag), resulting in the Al (111) peak and/or the Ag (111) peak at 38.8° and 38.3°, respectively. These two peaks are very close to each other and difficult to differentiate. When measured *via* SEM/EDS, the FCC phase is confirmed to be pure Al. In addition, the Ag_2_Al phase can be indexed *via* the peak at 41.5°. The inevitable Al_2_O_3_ surface oxide is always seen for films with a high Al content, with peaks at 37.1° and 52.2°. There seems to be a very minor Ag_2_O peak at about 55.5°.

### HCl in supercritical fluid

**Fig. 2:**
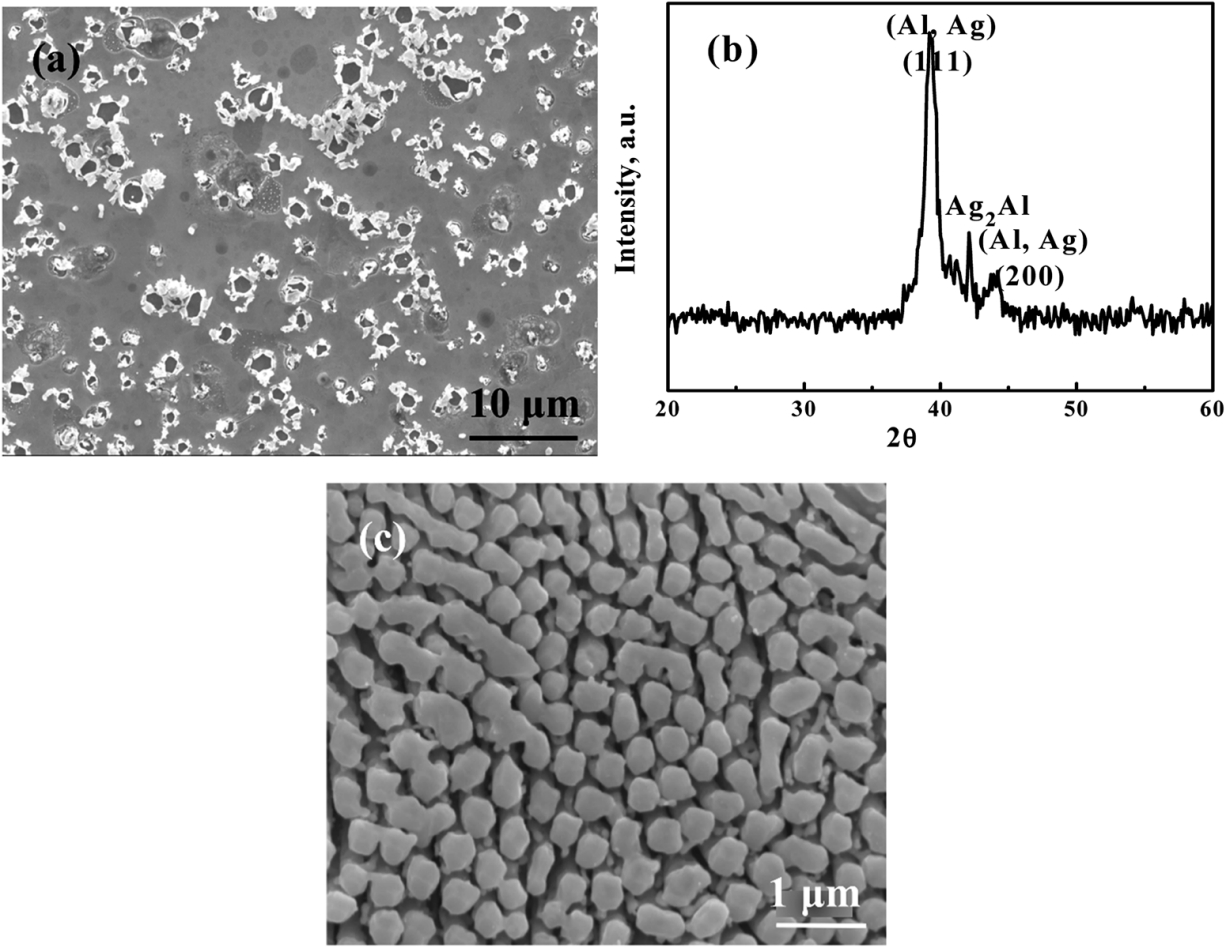
(a) An SEM/SEI micrograph of AgAl thin film dealloyed using 0.05 ml of 1 M HCl in supercritical CO_2_ for 3 min and (b) the associated XRD pattern, and (c) an SEM/SEI micrograph of film dealloyed *via* conventional chemical dealloying, using 5 wt% HCl aqueous solution first at 60 °C for 30 min until no obvious bubbles emerged, followed by continuous dealloying in the same solution at 90 °C for 120 min in order to further leach out residual Al in the samples.

From previous results,^2,10–13^ we know that HCl is an appropriate acid for selective etching in conventional chemical dealloying. But using HCl under SCF, the problem is the surface tension, which causes devastation of the silver ligaments. In this study, we added a very small amount (only 0.05 ml) of 5 wt% or 1 M HCl to the reaction chamber full of supercritical CO_2_, termed HC-SC. The results are quite similar, independent of HCl concentration. As shown in Fig. 2(a), a porous structure can be fabricated. But the pore size is about 2 μm to 7 μm, with an average of about 4 μm (Table 1), which is far larger than what we expected. The associated XRD pattern for the HC-SC dealloyed film in Fig. 2(b) indicates that the Ag_2_Al phase is still there: the dealloying is efficient.

**Table 1:** Results from Ag_35_Al_65_ samples dealloyed with 0.05 ml of 5 wt% or 1 M HCl in supercritical CO_2_ (termed HC-SC) at 120 °C and 2000 psi, for 3 and 5 min

	Pre-dealloyed	Dealloyed using HC-SC at 120 °C and 2000 psi for 3 min	Dealloyed using HC-SC at 120 °C and 2000 psi for 5 min
EDS results	Ag_35_Al_65_	Ag_35_Al_65_	Ag_36_Al_64_
Surface area (m^2^ g^−1^)	0	0.5	1.3
Porosity	0%	10%	15%
Average pore size (nm)	—	∼4000	∼4000

Table 1 also lists the EDS results for the thin film dealloyed using 0.05 ml of 5 wt% or 1 M HCl in supercritical CO_2_. The Ag and Al composition did not really change; the corrosion process is obviously not selective etching. The HCl in the SC reaction chamber is too aggressive and too corrosive; both Ag and Al are eaten. The surface area values for HC-SC dealloyed Ag_35_Al_65_ for 3 and 5 min, determined using BET, are 0.5 and 1.3 m^2^ g^−1^, respectively, and the porosity volume fraction values are 10% and 15%, respectively.

The reason for these circumstances is that HCl was not fully dissolved in the supercritical CO_2_. The supercritical fluid is known as a good solvent, and additive water or acid can act as a solute, forming a solution. The organic compound has low polarity, which makes it easier to dissolve in supercritical CO_2_. This is the reason why organic compounds like H_2_C_2_O_4_ can be fully dissolved in a supercritical fluid. On the contract, HCl has higher polarity. In the reaction chamber, it was not fully separated. Instead, the HCl liquid assembled and directly reacted with the thin film surfaces, including Ag and Al. As a result, we can conclude that if we want to fabricate a homogeneous and non-devastated nanoporous structure using supercritical fluid, using an organic acid that can be fully dissolved in the supercritical fluid is a suitable choice.

### Comparison between conventional chemical and supercritical fluid dealloying with HCl

Previous researchers used different inorganic acid and organic acids to dealloy. In this research, we intend to compare the results while using 5 wt% HCl typical chemical dealloying and 5 wt% HCl in supercritical fluid. Using HCl is the most common way to fabricate nanoporous silver from AgAl thin film. The reaction of Al in HCl (and the water in dilute HCl solution) is described below:^28^

1 2Al + 6H_2_O → 2Al(OH)_3_ + 3H_2_

2 Al(OH)_3_ + HCl → Al(OH)_2_Cl + H_2_O

Herein the generation of Al(OH)_2_Cl is important; it makes the reaction able to proceed quickly. The aqueous solution of 5 wt% HCl has higher surface tension than supercritical CO_2_. This would cause the solution to stay on the surface of the AgAl thin film for too long. HCl could not go through the tunnels between the ligaments. Instantly, it starts to etch Ag on the surface, as seen from Fig. 2(a). The reaction between HCl and Ag_2_Al is:

3 2Ag_2_Al + 3H_2_O → Al_2_O_3_ + 4Ag + 3H_2_

4 2Ag_2_Al + 6Cl^−^ + 3H_2_O → Al_2_O_3_ + 4Ag + 6HCl

5 2Ag + 2HCl → 2AgCl + H_2_

In contrast, the microstructure of a sample dealloyed with 5 wt% HCl using a typical chemical dealloy process is finer and more homogeneous, as shown in Fig. 2(c).

### Water in a supercritical fluid

**Fig. 3:**
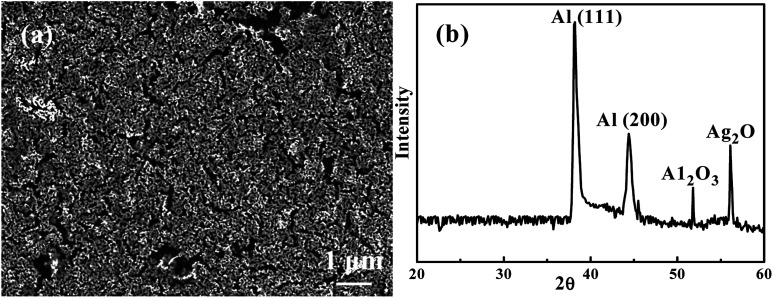
(a) An SEM/SEI micrograph of Ag_35_Al_65_ thin film after being dealloyed using SC water for 60 min, and (b) the associated XRD pattern.

Since dealloying with HCl in SCF was too corrosive, water in SCF was tried. Fig. 3(a) shows an SEM secondary electron image (SEI) of an Ag_35_Al_65_ thin film dealloyed using water in supercritical CO_2_ for 60 min, termed as W-SC. There are many ligaments on the surface. The distance between ligaments, equivalent to the pore size, is from 100 to 300 nm, with an average value of ∼200 nm. Fig. 3(b) is the associated XRD pattern of W-SC after 60 min. The signal intensity of Ag_2_Al has decreased appreciably, while Al (111) and Al (200) signals are still strong. The corrosion started with the intermetallic compound Ag_2_Al, because it has the higher potential energy. After corrosion, Ag_2_O would form, showing a peak at 56.5°.

Water has high solubility in supercritical CO_2_, and both water and CO_2_ are common materials we can obtain from nature. Normal reverse osmotic water has carbonate (CO_3_^2−^) inside because a small amount of CO_2_ gas in the atmosphere dissolves in water. The water has strong oxidative capacity in supercritical CO_2_, which could react with alumina. The alumina ions will hydrolyze because of the presence of carbonate, and form aluminum carbonate. Aluminum carbonate will form aluminum hydroxide after reacting with water. Therefore, the reaction of Ag–Al film in SC-W is proposed below:

6 Al + H_2_O →Al_2_O_3_ + H_2_

7 2Al + 6H_2_O → 2Al(OH)_3_ + 3H_2_

8 Al_2_O_3_ + CO_3_^2−^ → Al_2_(CO_3_)_3_

9 Al_2_(CO_3_)_3_ + H_2_O → 2Al(OH)_3_ + 3CO_2_

10 Al(OH)_3_ + 3H^+^ → Al^3+^ + 3H_2_O

As for the Ag_2_Al part, it should proceed as follows:

11 2Ag_2_Al + 3H_2_O → Al_2_O_3_ + 4Ag + 3H_2_

12 2Ag_2_Al + H_2_CO_3_ + 3H_2_O → Al_2_(CO_3_)_3_ + 4Ag + 3H_2_

13 Ag + H_2_O → Ag_2_O + H_2_

Table 2 summarizes the W-SC results. With supercritical CO_2_ mixed with water, we could dealloy an Ag–Al alloy, and fabricate nanoporous silver thin film. The surface area values for W-SC dealloyed Ag_35_Al_65_ for 30 and 60 min, determined using BET, are 1.9 and 2.5 m^2^ g^−1^, respectively, and the porosity volume fraction values are 25% and 32%, respectively. The reason for the lower porosity fraction might be due to the atomic percent of Ag in the Ag_35_Al_65_ thin film being only 35 at%. If we increase the percentage of Ag, it will fabricate a sponge structure with smaller pore sizes. Overall, W-SC dealloying appears to perform better than HC-SC, but the efficiency after 60 min is still below expectations. The third attempt was applying H_2_C_2_O_4_.

**Table 2:** Results from Ag_35_Al_65_ samples dealloyed with water in supercritical CO_2_ (termed W-SC) at 120 °C and 2000 psi, for 30 and 60 min

	Pre-dealloyed	Dealloyed using W-SC at 120 °C and 2000 psi for 30 min	Dealloyed using W-SC at 120 °C and 2000 psi for 60 min
EDS results	Ag_35_Al_65_	Ag_53_Al_47_	Ag_59_Al_41_
Surface area (m^2^ g^−1^)	0	1.9	2.5
Porosity	0%	25%	32%
Average pore size (nm)	—	∼200	∼200

Note that water alone cannot dealloy AgAl using a conventional chemical dealloying method. Therefore, no comparison can be made regarding the dealloying differences between conventional chemical dealloying and supercritical fluid dealloying.

### H_2_C_2_O_4_ in supercritical fluid

**Fig. 4:**
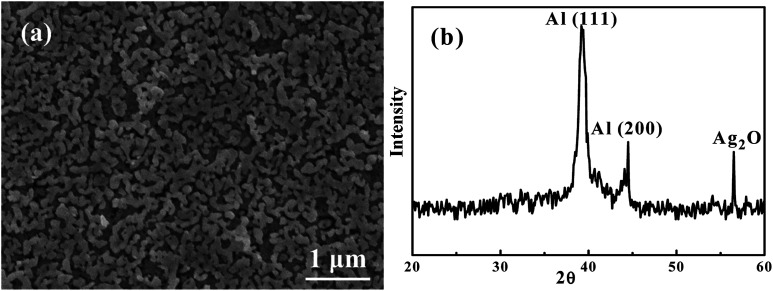
(a) An SEM/SEI micrograph of Ag_35_Al_65_ thin film after being dealloyed using SC H_2_C_2_O_4_ at 2000 psi for 20 min, and (b) the associated XRD pattern.

Fig. 4(a) is an SEI image showing the microstructure of Ag_35_Al_65_ thin film after it was dealloyed using 0.25 ml of 5 wt% H_2_C_2_O_4_ in supercritical fluid CO_2_, termed HCO-SC. Compared with the thin film dealloyed using water in supercritical fluid for 60 min, the distance between ligaments, equivalent to the pore size, in the HCO-SC film is smaller (∼100 nm *versus* ∼200 nm). The associated XRD pattern of the Ag_35_Al_65_ thin film after it was dealloyed by HCO-SC is presented in Fig. 4(b). The Ag_2_Al signal is completely gone, and the signals of Al (111) and Al (200) have decreased a lot compared with those of the as-sputtered thin film in Fig. 1(b).

The water in the H_2_C_2_O_4_ dilute solution mixed with supercritical CO_2_ has strong oxidizing abilities. The strong oxidizing abilities oxidize aluminum to Al(OH)_3_, which reacted with H_2_C_2_O_4_ and formed Al_2_(C_2_O_4_)_3_. Al_2_(C_2_O_4_)_3_ can be dissolved and taken away with the supercritical fluid when the experiment is over. According to Kotz *et al.*,^28^ the reaction between Al, H_2_C_2_O_4_ and water (within the H_2_C_2_O_4_ dilute solution) proceeds as presented below:

14 2Al + 6H_2_O → 2Al(OH)_3_ + 3H_2_

15 2Al(OH)_3_ + 3H_2_C_2_O_4_ → Al^3+^ + C_2_O_4_^2−^ + 6H_2_O

As for the Ag_2_Al part, this should proceed as follows:

16 2Ag_2_Al + 3H_2_O → Al_2_O_3_ + 4Ag + 3H_2_

17 2Ag_2_Al + C_2_O_4_^2−^ + H_2_O → Al_2_O_3_ + 4Ag + H_2_C_2_O_4_

18 Ag + H_2_O → Ag_2_O + H_2_

Table 3 lists the EDS results for the dealloyed thin films. The thin film, after being dealloyed under conditions of 120 °C and 2000 psi, shows an obvious drop in Al content from 65% down to 25% in 10 min, and then to 12% in 20 min. This proves that using supercritical CO_2_ as the solvent really increases the selective etching of an AgAl alloy. The surface area values for HCO-SC dealloyed Ag_35_Al_65_ for 10 and 20 min, determined using BET, are 2.9 and 4.5 m^2^ g^−1^, respectively, and the porosity volume fraction values are 37% and 72%, respectively.

**Table 3:** Results from Ag_35_Al_65_ samples dealloyed using 1 ml of H_2_C_2_O_4_ in supercritical CO_2_ (termed HCO-SC) at 120 °C and 2000 psi, for 10 and 20 min

	Pre-dealloyed	Dealloyed using HCO-SC at 120 °C and 2000 psi for 10 min	Dealloyed using HCO-SC at 120 °C and 2000 psi for 20 min
EDS results	Ag_35_Al_65_	Ag_75_Al_25_	Ag_88_Al_12_
Surface area (m^2^ g^−1^)	0	2.9	4.5
Porosity	0%	37%	72%
Average pore size (nm)	—	∼100	∼100

### H_2_C_2_O_4_ in supercritical fluid for annealed samples

After physical vapor deposition, an AgAl alloy sputtered thin film on a silicon substrate can be loose in some locations, and Ag and Al might not be co-sputtered completely uniformly. A post-sputtering annealing process might be a method to improve this problem. We thus place the Ag_35_Al_65_ films in an annealing chamber and conduct annealing at 400 °C for 20 min under an Ar atmosphere.

**Fig. 5:**
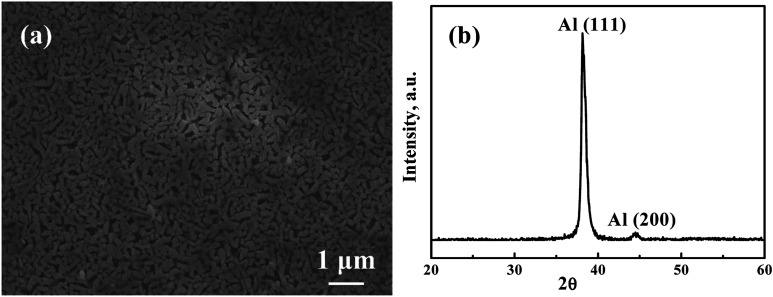
(a) An SEM/SEI micrograph of annealed Ag_35_Al_65_ thin film dealloyed using SCF with 1 ml of 5 wt% H_2_C_2_O_4_ under conditions of 120 °C and 2000 psi for 20 min, and (b) the associated XRD pattern.

Fig. 5(a) is the annealed Ag_35_Al_65_ thin film dealloyed using 0.25 ml of 5 wt% H_2_C_2_O_4_ in supercritical fluid CO_2_ under the conditions of 120 °C and 2000 psi. The ligament size is about 150 nm, and the pore size is 50 nm to 100 nm, with an average size of ∼75 nm. The XRD scan of the annealed and dealloyed samples is basically the same as that in Fig. 5(b). Note that there are no peaks relating to Al_2_O_3_ or Ag_2_O. The pore size is smaller than any other research reported. Table 4 shows the EDS results for the annealed thin film. From the atomic percentages, it is obvious that Al has been selectively etched. Most importantly, there is no signal from oxygen. It seems that the oxygen trapped in the sputtered films might have been released from the films into the Ar atmosphere during annealing. Normally, the dealloying process in supercritical fluid will lead to the formation of some metallic oxides. But the oxides become minimal in the annealed and then dealloyed films, consistent with the findings from XRD, as shown in Fig. 5(b). The surface area values for HCO-SC annealed and then dealloyed Ag_35_Al_65_ for 10 and 20 min, determined using BET, are 3.7 and 4.6 m^2^ g^−1^, respectively, and the porosity volume fraction values are 50% and 74%, respectively.

**Table 4:** Results from samples annealed for 20 min dealloyed using 1 ml of 5 wt% H_2_C_2_O_4_ in supercritical fluid CO_2_ (termed HCO-SC) at 120 °C and 2000 psi, for 10 and 20 min

	Pre-dealloyed	Sample annealed for 20 min dealloyed using HCO-SC at 120 °C and 2000 psi for 10 min	Sample annealed for 20 min dealloyed using HCO-SC at 120 °C and 2000 psi for 20 min
EDS results	Ag_35_Al_65_	Ag_80_Al_20_	Ag_90_Al_10_
Surface area (m^2^ g^−1^)	0	3.7	4.6
Porosity	0%	50%	74%
Average pore size (nm)	—	∼75	∼75

### Comparison between conventional chemical and supercritical fluid dealloying using H_2_C_2_O_4_

**Fig. 6:**
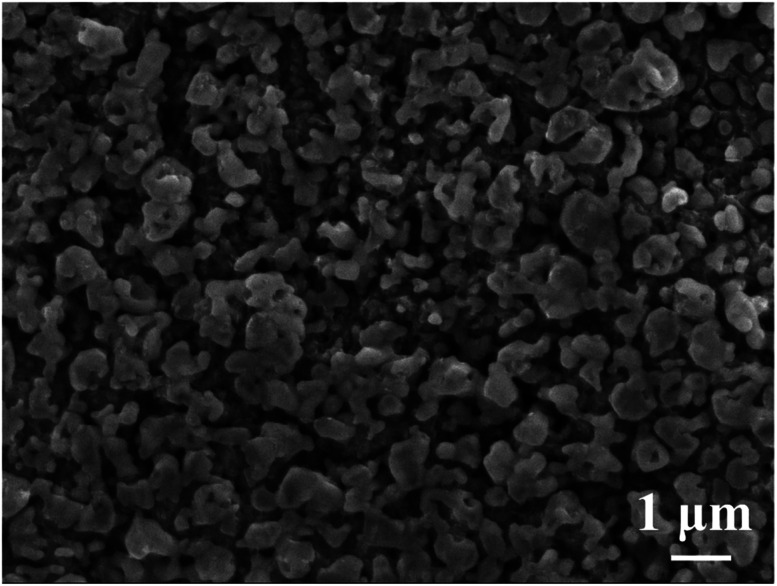
An SEM/SEI micrograph showing the conventional chemical dealloying microstructure of Ag_35_Al_65_ dealloyed with 10 wt% H_2_C_2_O_4_ for 60 min to form the nanoporous structure.

From the SEM image in Fig. 6, typical chemical dealloying using 10 wt% H_2_C_2_O_4_ could form a nanoporous structure within 60–600 min. And from higher magnifications, we can discover that the pore size varies from 100 nm to 1000 nm, which is non-uniform.

On the other hand, the alloy dealloyed using supercritical fluid only needs to use a very small amount of chemical, 0.25 ml, in SCF for only 20 min to achieve a fully etched pore structure, as shown in Fig. 4(a) and 5(a). From the SEM image, we found that the pore size is uniform and much smaller than the one obtained upon typical chemical dealloying with H_2_C_2_O_4_. The promising nanoporous structures are well demonstrated.

### Closing remarks

For an overall comparison of conventional and supercritical dealloying, the benefits of applying the latter can be demonstrated by the data listed in Table 5. Conventional chemical dealloying can be done using HCl, NaOH, H_2_C_2_O_4_, HSO_4_, *etc.* The chemical usually needs to be present in the amount of a few liters, with a chemical concentration in the level of 5–10 wt%. The time required to complete dealloying would range from 30 to 600 min.

**Table 5:** Comparison between conventional chemical and supercritical dealloying of an Ag–Al system

Method	Chemical	Concentration	Time (min)	Reference
Conventional	HCl	5 wt%	30–120	2009 (ref. 10)
	HCl	5 wt%	150	2015 (ref. 11)
	HCl	5 wt%	60	Current study
	NaOH	20 wt%	30–120	2009 (ref. 10)
	H_2_C_2_O_4_	10 wt%	600	2011 (ref. 12)
	H_2_C_2_O_4_	10 wt%	60	Current study
	HSO_4_	5 wt%	600	2011 (ref. 12)
Supercritical	HCl	0.05 ml 5 wt% in SCF	3	Current study
	Water	5 ml in SCF	60	Current study
	H_2_C_2_O_4_	0.25 ml 5 wt% in SCF	20	Current study

On the other hand, supercritical fluid dealloying requires the chemical in a much smaller amount, from 0.05 to 0.25 ml, with the chemical concentration comparable or lower, and the dealloying time period appreciably shortened down to about 20 min. Even plain water in SCF exposes selective dealloying capabilities. Furthermore, the resulting open-cell pores can be highly uniform and the pore size can be apparently smaller, down to ∼75 nm.

## 4. Conclusions

Based on the current study, the following conclusions can be drawn: (1) when using a supercritical fluid as the solvent in the dealloying corrosion process, it takes much less time and a lower chemical concentration than traditional selective etching and electrochemistry. The SCF performs much better in an environmentally friendly matter in fabricating nanoporous structures for catalysts, optics, sensors, filters, *etc.*; (2) it is found that a higher temperature and pressure would accelerate SCF dealloying. In this study, it is found that 120 °C and 2000 psi would be a suitable combination; (3) under normal temperatures and pressures, water does not have the ability to dealloy into a nanoporous structure. But in supercritical CO_2_, with higher temperature and higher pressure, water has the high oxidizing ability to do so; (4) according to previous research, H_2_C_2_O_4_ is a suitable acid to form nanoporous silver, but it would need 60–600 min to result in successful dealloying, and the concentration needs to be as high as 10 wt%. Now, using supercritical CO_2_ as a solvent, we only need 0.25 ml of 5 wt% H_2_C_2_O_4_ and 20 min to form a nanoporous structure. The average pore size can be as low as 75 nm, which is much smaller and more uniform than in structures prepared using conventional chemical dealloying. The benefits of applying SCF are apparent and well demonstrated.

## Conflicts of interest

There are no conflicts to declare.

## Supplementary Material
